# Trends and correlates of overweight/obesity in Czech adolescents in relation to family socioeconomic status over a 12-year study period (2002–2014)

**DOI:** 10.1186/s12889-017-5013-1

**Published:** 2018-01-10

**Authors:** Erik Sigmund, Petr Badura, Dagmar Sigmundová, Jaroslava Voráčová, Jiří Zacpal, Michal Kalman, Jan Pavelka, Jana Vokacová, Vladimír Jr Hobza, Zdenek Hamrik

**Affiliations:** 1 Institute of Active Lifestyle, Faculty of Physical Culture, Palacký University Olomouc, Tr. Miru 117, 77111 Olomouc, Czech Republic; 2 Department of Social Sciences in Kinanthropology, Faculty of Physical Culture, Palacký University Olomouc, Tr. Miru 117, 77111 Olomouc, Czech Republic; 3 Department of Recreation and Leisure Studies, Faculty of Physical Culture, Palacký University Olomouc, Tr. Miru 117, 77111 Olomouc, Czech Republic; 4 Department of Computer Science, Faculty of Science, Palacký University Olomouc, Tr. 17. Listopadu, 77146 Olomouc, Czech Republic

**Keywords:** Trends, Family affluence, Excessive body weight, Moderate-to-vigorous physical activity, Screen time, Adolescents, HBSC questionnaire

## Abstract

**Background:**

This study examined a) trends in overweight/obesity, moderate-to-vigorous physical activity (MVPA), and screen time (ST) among Czech adolescents over a 12-year study period (2002–2014) in relation to family affluence (FA) and b) correlates of adolescent overweight/obesity from different FA categories.

**Methods:**

A nationally representative sample of 18,250 adolescents (51.4% girls) aged 10.5–16.5 years was drawn from the Czech Health Behaviour in School-aged Children questionnaire-based surveys in 2002, 2006, 2010, and 2014. Using the FA scale, the socioeconomic status (SES) of the respondents’ families was assessed. SES-stratified trends in the prevalence of overweight/obesity meeting the MVPA (≥60 min/day), and ST (≤2 h/day) recommendations were assessed using logistic regression.

**Results:**

A trend-related significant increase (*p* < 0.05) in the prevalence of overweight/obesity was observed in low−/medium-FA boys and medium−/high-FA girls. Unlike in high-FA adolescents, a significant decrease was revealed in the rates of meeting the MVPA recommendation in low-FA boys (28.9%_2002_ → 23.3%_2014_, OR = 0.75, 95% CI = 0.59–0.95, *p* < 0.05) and girls (22.3%_2002_ → 17.3%_2014_, OR = 0.73, 95% CI = 0.57–0.92, *p* < 0.01). A significant (*p* < 0.001) trend-related increase in excessive ST was evident in adolescents regardless of gender and FA category. Generally, girls and older adolescents had lower odds of overweight/obesity than boys and 11-year-old adolescents. While in the high-FA category of adolescents, achieving 60 min of MVPA daily and the absence of excessive ST on weekdays significantly (*p* < 0.01) reduced their odds of being overweight/obese, in low-FA adolescents this was not the case.

**Conclusions:**

High rates of overweight/obesity and a poor level of daily MVPA among low-FA children provide disturbing evidence highlighting the necessity of public health efforts to implement obesity reduction interventions for this disadvantaged population.

## Background

Obesity and obesity-related diseases are among the most expensive economically treatable diseases in children and youth [1, 2]. After years of a consistent increase in the prevalence of childhood obesity, there are some indices of a possible decline in the prevalence of obesity among pre-schoolers, children, and adolescents [3–5]. However, this ‘break’ in the obesity epidemic needs to be interpreted with caution [6], since the prevalence of extreme forms of obesity in children and adolescents continues to increase [7, 8]. In addition, in economically developed countries the peak prevalence of obesity is moving from middle adult age to younger age cohorts [9]. Childhood obesity is the focus of public health efforts and the accurate determinate of obesity-vulnerable groups of children and youth is a key condition for the development and implementation of obesity reduction interventions. Age, gender, socioeconomic status (SES), ethnicity, level of physical activity (PA) and sedentary behaviour, eating habits, the duration of sleep, and behavioural problems should be taken into account [10, 11].

Repeatedly observed positive links between childhood overweight/obesity and unhealthy eating habits [12–14] or low PA or high sedentary behaviour [15–19] are influenced by the SES of children’s families [20, 21]. SES in children and adolescents is commonly estimated using parental education or parental income [15, 21]. In the high-income countries (e.g. the U.S., Germany) high levels of adolescents’ PA and participation in sport are significantly associated with a higher level of parental education [15, 21, 22], family income [15, 21], and the adolescents’ level of education [23], regardless of their gender [15, 21]. Moreover, European adolescents with parents with lower levels of education spend more time on sedentary activities than adolescents whose parents have higher education [24]. Similarly, children from families with a low household income show lower levels of PA and spend more time being sedentary than children from families with a high household income [25, 26]. However, at the beginning of the third millennium (2001/2002) the effect of family affluence (FA) was significant in relation to MVPA neither in Czech adolescents nor in adolescents from other middle- or low-income European countries – Greenland, Ireland, Malta, Ukraine, and Macedonia [27].

Probably, adolescents from low- and middle-income European countries tend to repeat behavioural patterns that had previously been witnessed in adolescents from the Western high-income countries, e.g. a decrease in PA, increased sedentary behaviour, screen-based activities, and an increase in the excessive consumption of sweetened beverages and fast food intake [28, 29], which consequently leads to increased rates of overweight and obesity [10–12]. In addition, across the low- and middle-income countries of Eastern, Southern, and Central Europe (e.g. Belarus, Latvia, the Republic of Moldova, Croatia, Greece, Malta, Romania, Serbia, Slovenia, the Czech Republic, and Slovakia) a rapid increase in overweight/obesity and obesity-related diseases has been projected to occur by 2030 [30] or by 2050 [30, 31]. Moreover, out of the above-mentioned European countries, the largest reduction in diabetes and coronary heart diseases, with a 5% fall in the body mass index (BMI) of the population by 2030, is modelled in Croatia, the Czech Republic, Latvia, Serbia, and Slovakia [30, 31]. Therefore, it is necessary to improve the surveillance of trends in obesity, as well as obesity-related practices that are effective in reducing body fat in low- and middle-income European countries.

Previous ‘Health Behaviour in School-aged Children’ (HBSC) trend studies point to an increase in the prevalence of overweight/obesity among adolescents from low- and middle-income European countries between 2002 and 2010 [32, 33], which, in the Czech Republic, continued until 2014 [16]. In the 2002–2010 period, mixed results were found in trends to achieve of at least 60 min of MVPA per day in adolescents from low- and middle-income European countries [34]. However, the same pattern of behaviour in adolescent girls and boys was found in screen time (ST) trends between 2002 and 2010 – a slight decline in television viewing (TV), more than offset by a sharp increase in computer use (PC) [35]. Moreover, excessive ST behaviour (more than two hours per day) and insufficient PA (less than 60 min of MVPA per day) significantly increase the chance of overweight/obesity in Czech adolescents [16]. However, it is still not known whether the trends in overweight/obesity, PA, and ST vary according to the SES of children’s families.

Therefore, the main aim of this study is to describe trends in the prevalence of overweight/obesity and in achieving MVPA recommendations (≥60 min per day) and non-excessive ST (≤2 h per weekday or weekend day) among Czech boys and girls over a 12-year study period (2002–2014) in relation to the SES of their families. Furthermore, the study aims to analyse the correlates of overweight/obesity in adolescents from different SES categories.

## Methods

### HBSC study

The HBSC study is a World Health Organization (WHO) collaborative cross-national study carried out by research teams in 47 countries and regions across Europe, North America, and Asia. It aims to uncover adolescents’ health and health-related behaviours, and provides insights into the adolescents’ lifestyle. A standardized international research protocol was followed in all the participating countries to ensure consistency in survey instruments, data collection, and processing procedures [36]. Since 1985/1986 data has been collected at four-year intervals using nationally representative samples of 11-, 13-, and 15-year-old adolescents within each participating country. The questionnaire survey assesses a wide range of self-reported health behaviours and lifestyle-related outcomes.

### Sample and data collection

The data for the present analyses is based on a nationally representative randomized cluster (school level) sampling procedure. The sample consists of reports from 18,250 adolescents (2002: *n* = 4912 (52.3% girls), 2006: *n* = 4629 (49.6% girls), 2010: *n* = 4121 (51.3% girls), and 2014: *n* = 4588 (52.4% girls)) with complete data for weight status, PA, ST, and FA variables (Table 1). The trained researchers administered the data collection during one morning teaching lesson in the classroom. The participation of the adolescents in each data collection cycle was voluntary, and the respondents were assured of anonymity and the confidentiality of their responses. To ensure anonymity, after completing the survey the students were instructed to insert the questionnaire in an envelope, seal it, and hand it over to the researchers. The school response rate among the surveys varied from 75% to 99% and the adolescent participants’ response rate in each of the four surveys exceeded 80%. Participating adolescents were predominantly white Caucasian (>98%), which is representative of the ethnic demographics of the Czech Republic [37]. All the survey procedures for each data collection cycle were documented and can be downloaded from http://www.hbsc.org/methods/index.html. This study was approved by the Institutional Research Ethics Committee of the Faculty of Physical Culture, Palacký University Olomouc, No. 9/2016 on 4th March 2016.

**Table 1:** Descriptive characteristics of the samples, HBSC study, Czech Republic 2002–2014

	2002		2006		2010		2014	
	Boys	Girls	Boys	Girls	Boys	Girls	Boys	Girls
	(*n*=2345)	(*n*=2567)	(*n*=2332)	(*n*=2297)	(*n*=2008)	(*n*=2113)	(*n*=2183)	(*n*=2405)
	%	%	%	%	%	%	%	%
Chronological age^a^								
11 years	34.1	33.1	31.4	31.2	32.6	30.6	29.9	30.3
13 years	32.5	33.8	33.5	33.9	31.4	35.2	33.9	34.4
15 years	33.4	33.1	35.0	34.9	36.0	34.2	36.2	35.3
Family affluence								
Low	34.2	40.5	25.7	30.3	13.1	17.0	32.4	34.4
Medium	56.5	53.0	54.9	55.9	54.2	53.3	44.3	45.4
High	9.3	6.5	19.4	13.8	32.7	29.7	23.3	20.2
Weight status								
Normal weight	81.6	91.7	78.0	83.8	73.8	88.1	74.9	87.9
Overweight/obese	18.4	8.3	22.0	16.2	26.2	11.9	25.1	12.1
Daily MVPA								
≥ 60 min	32.2	23.1	27.6	18.0	28.1	18.9	25.6	18.9
Daily screen time								
> 2 h per weekday	75.3	85.9	87.9	88.8	61.3	73.4	81.7	77.0
> 2 h per weekend day	78.3	65.0	88.6	79.8	89.8	84.7	91.7	84.0

MVPA – moderate-to-vigorous physical activity; Screen time – amount of time spent watching television or using a computer; ^a^ 11 years (13 years and 15 years) includes adolescents in the age range 10.5–12.49 years (12.50–14.49 years and 14.50–16.49 years)

### Survey items

#### Weight status

Three weight status-related items (body weight, body height, and chronological age) were used to calculate the prevalence of normal body weight and overweight/obesity of the participants. The actual body weight and height of the adolescents were self-reported in the HBSC questionnaire. The chronological age was calculated as the difference between the date of the data collection and the self-reported month and year of birth of the participating adolescents. Body weight status (normal weight and overweight/obesity) was classified on the basis of the WHO percentile Body Mass Index (BMI) charts for 5–19-year-old boys and girls [38]. Overweight and obesity were represented by 85%–97% and >97%, respectively, on age-differentiated percentile BMI charts [39, 40]. The BMI derived from the self-reported body weight and height demonstrated good diagnostic ability for identifying overweight/obesity in the 6–18-year-old children (sensitivity, specificity, and positive and negative predictive values ranged from 0.83 to 0.98) [41]. Self-reported body weight and body height demonstrated almost perfect agreement with the measured values and were substantially able to identify overweight-to-obese children in epidemiological studies [41, 42].

#### Physical activity

MVPA patterns was assessed for the purposes of analyses of trends. MVPA was examined by a single question, ‘*Over the past 7 days, on how many days were you physically active for a total of at least 60 minutes per day?*’ The question was introduced by an explanatory text defining MVPA as any activity that increases the heart rate and makes a person get out of breath some of the time, with examples provided. The response categories were consistent among all the survey cycles and ranged from ‘*0 days’* to ‘*7 days’*. For the analysis of trends in achieving current MVPA recommendations (≥60 min per day – [43, 44]) a dichotomous outcome variable was created. Adolescents who self-reported that they had been active for at least 60 min of MVPA on each of the past seven days were classified as meeting the MVPA recommendations.

The assessment of self-reported MVPA during the past seven days in adolescents was originally developed and validated against seven-day continuous measurement with an accelerometer (r_MVPA_ = 0.40 *p* < 0.01) [45]. Recent studies support the validity of self-reported past-seven-days total PA and MVPA (*r* = 0.49 p < 0.01 correlation with seven-day continuous monitoring with an Actigraph accelerometer) [46], with almost perfect test-retest stability of the MVPA item in 11–15-year-old Polish (ICC = 0.98) and Chinese (ICC = 0.82) adolescents [47, 48].

#### Screen time

Two ST-related components (TV viewing and PC use) were assessed in a comparable manner in all the four-year cycles of data collection between 2002 and 2014. TV viewing during leisure time was examined using one question divided into weekdays and weekends: ‘*About how many hours a day do you usually watch television (including DVDs and videos) in your free time?*’ The response options were consistent among all the data collection cycles and included nine response categories: ‘*None at all*’, ‘*About half an hour a day*’, ‘*About 1 h a day*’, ‘*About 2 h a day*’, ‘*About 3 h a day*’, ‘*About 4 h a day*’, ‘*About 5 h a day*’, ‘*About 6 h a day*’, and ‘*About 7 or more hours a day*’. In 2002, PC time was assessed by the question: ‘*About how many hours a day do you usually use a computer (for playing games, e-mailing, chatting, or surfing the Internet) in your free time?*’ During the 2006–2014 surveys, this single question was subdivided into two to better reflect the wider accessibility of new technologies. Adolescents were asked to report separately for weekdays and weekends: i) ‘*About how many hours a day do you usually use a computer for chatting online, Internet, e-mailing, homework, and so forth in your free time?*’; and ii) ‘*About how many hours a day do you usually play games on a computer or games console (PlayStation, Xbox, GameCube, etc.) in your free time?*’ Both PC time-related questions used the same categories of possible answers as TV viewing. The outcome variable, the amount of daily ST, is the sum of daily TV viewing and PC use. In line with the current recommendations [49], daily ST was dichotomized as follows: two or fewer hours vs. more than two hours of ST per day. Two or more hours per day of ST is classified as excessive [49].

The validity of self-reported ST questions for the past seven days has been proved in comparison with 7-day 24-h diaries among 11-to-15-year-old adolescents both on weekdays (*r* = 0.39–0.46, *p* < 0.001) and at weekends (*r* = 0.37–0.47, p < 0.001) [50, 51]. The test-retest stability of the TV viewing and PC use question has been repeatedly verified for weekdays (ICC_TV_ = 0.54–0.72, ICC_PC_ = 0.33–0.82) and weekend days (ICC_TV_ = 0.58–0.68, ICC_PC_ = 0.33–0.66) [47, 48, 50, 52]. Adolescents do not have a systematic tendency to under- or over-report their amount of daily ST, and the current TV and PC time-related questions appear to have adequate reliability and validity for behavioural epidemiological studies [47, 48, 50–52].

#### Family affluence scale

Because of the repeatedly uncovered gender-related differences in the prevalence of overweight/obesity [32], PA patterns [33, 34], and ST behaviour [35] of adolescents, all the trend analyses were stratified according to the gender of the adolescents. Parental SES has also been shown to correlate with teenagers’ weight status [11, 20], PA [25, 26], and ST [51], and therefore we stratified the analysis further by FA as an indicator of SES.

The FA scale was developed within the HBSC research network as a measure of parental SES [53]. The FA scale, including easily answerable questions that reflect material affluence, has proved to be a useful indicator of family material affluence [53]. Between 2002 to 2010, four items were included to determine FA: *car ownership* (No = 0; One = 1; Two or more = 3), *number of computers* (None = 0; One = 1; Two = 2; Three or more = 3), *family holidays in the past year* (Never = 0; Once = 1; Twice = 2; Three or more times = 3), and *having one’s own bedroom* (No = 0; Yes = 1) [53]. In 2014, the FA scale was updated with respect to the changes in the social environment [44, 54]. Two new FA-related items – *dishwasher ownership* (No = 0; Yes = 2) and *number of bathrooms* (None = 0; One = 1; Two = 2; Three or more = 3) were added to the existing FA scale. The response codes to these items were summed and treated as a composite sum score. For the trend analyses, three categories of FA (“low”, “medium”, and “high”) were derived from the composite sum score. Between 2002 and 2010 the FA categories correspond to tertiles of the sum score (“low” = 0–3; “medium” = 4–6, “high” = 7–9) and in 2014 as follows: “low” = 0–6, “medium” = 7–9, and “high” = 10–13 [14]. The FA scale provides a valid assessment of household material affluence among families with adolescents [53] with documented high validity (kappa coefficients 0.41%–0.74%, 76.2%–88.1% agreement) and moderate reliability (Cronbach’s α = 0.58) between children’s and parents’ responses on the FA scale-related items [55–58].

### Data analysis

Descriptive data for all the items analysed is presented as percentages split by gender per each survey year. Because TwoStep cluster analysis found no indicator for clustering by school or class in the outcome variables that were analysed in any of the four survey cycles, we conducted single-level analyses of the trend-related variables. The time trends of the outcome variables between 2002 and 2014 were determined using logistic regression analysis (Enter method), with the 2002 survey as the reference category. The chi-square tests compared the prevalence of overweight/obesity, prevalence in achieving MVPA, and ST recommendations in 2014 between groups of children with low and high FA separately for boys and girls. The correlates of overweight/obesity in relation to FA were estimated using the multiple logistic regression analysis Enter method. The regression parameters were based on the odds ratio (OR) with a 95% confidence interval (CI). The Statistical Package for the Social Sciences (SPSS) for Windows v.22 software (IBM Corp. Released 2013. Armonk, NY, USA) was used for data management and all statistical analyses. A minimum alpha level of 5% was taken for all the statistical analyses.

## Results

### Gender and family affluence-related trends in overweight/obesity, PA, and ST

A trend-related significant increase (>5 percentage points (p.p.)) in the prevalence of overweight/obesity was observed in low−/medium-FA boys and medium−/high-FA girls (Table 2). The highest prevalence of overweight/obesity in the 2013/2014 HBSC data collection was observed in the category of low-FA adolescents (boys 29.0% and girls 14.4%). Conversely, the lowest prevalence of overweight/obesity in 2014 was found in boys (20.1%) and girls (9.1%) in the high-FA category (Table 2). The differences in the prevalence of overweight/obesity in adolescents from the low- and high-FA categories were a significant for both genders in 2014.

**Table 2:** Trends in prevalence of overweight/obesity, PA and ST recommendations in relation to gender and FA in Czech adolescents: HBSC survey 2002–2014

Odds ratio to reach the variables^1–5^		2002	2006	2010	2014		2014	vs	2002^§^
								95%	CI
		%^a^	%^a^	%^a^	%^a^	p.p.	OR	Lower	Upper
Overweight/obese^1^									
*Boys*	Low FA	19.7	22.2	23.9	°29.0^###^	+9.3%	1.67***	1.32	2.12
	Medium FA	18.0	22.5	27.6	24.9	+6.9%	1.51***	1.23	1.85
	High FA	16.1	20.6	24.8	°20.1	+4.0%	1.31	0.86	1.99
*Girls*	Low FA	11.5	15.1	14.2	°14.4^##^	+2.9%	1.30	0.99	1.70
	Medium FA	6.5	17.5	11.0	11.6	+5.1%	1.90***	1.43	2.53
	High FA	4.2	13.2	12.1	°9.1	+4.9%	2.30*	1.02	5.21
MVPA recommendation^2^									
*Boys*	Low FA	28.9	23.5	27.8	°23.3	−5.6%	0.75*	0.59	0.95
	Medium FA	34.2	26.3	25.0	24.5	−9.7%	0.63***	0.52	0.76
	High FA	32.2	36.6	33.2	°30.9^##^	−1.3%	0.94	0.67	1.32
*Girls*	Low FA	22.3	14.8	17.9	°17.3	−5.0%	0.73**	0.57	0.92
	Medium FA	23.7	18.5	18.2	17.9	−5.8%	0.70**	0.58	0.86
	High FA	23.2	22.8	20.7	°23.7^##^	+0.5%	1.03	0.68	1.57
Non-excessive ST weekday^3^									
*Boys*	Low FA	28.2	17.3	16.7	°12.1^#^	−16.1%	0.35***	0.27	0.46
	Medium FA	23.5	13.7	10.5	11.6	−11.9%	0.43***	0.34	0.54
	High FA	19.4	10.8	13.0	°9.1	−10.3%	0.42***	0.26	0.65
*Girls*	Low FA	39.5	27.9	19.2	°23.6	−15.9%	0.47***	0.39	0.58
	Medium FA	37.6	25.8	18.9	21.6	−16.0%	0.46***	0.38	0.55
	High FA	42.9	27.1	16.7	°25.2	−17.7%	0.45***	0.31	0.65
Non-excessive ST weekend^4^									
*Boys*	Low FA	23.7	13.0	11.8	°9.0	−14.7%	0.32***	0.24	0.43
	Medium FA	20.7	10.8	10.0	8.4	−12.3%	0.35***	0.27	0.46
	High FA	20.9	11.1	9.9	°7.1	−13.8%	0.29***	0.18	0.46
*Girls*	Low FA	38.0	23.0	16.7	°18.3^#^	−19.7%	0.37***	0.29	0.45
	Medium FA	32.6	18.5	15.9	15.3	−17.3%	0.37***	0.31	0.46
	High FA	35.7	20.6	13.5	°13.8	−21.9%	0.29***	0.19	0.43

*FA* family affluence, *MVPA* moderate-to-vigorous physical activity, *ST* screen time; p.p. – percentage points between 2002 and 2014 (2006 and 2014 for VPA recommendation); OR – odds ratio; CI – 95% confidence interval; logistic regression Enter method (LR): ^1^OR of being overweight/obese, ^2^OR of achieving MVPA recommendations (≥60 min of MVPA per day), ^3,4^OR of meeting ST recommendations (≤2 h per weekday or ≤2 h per weekend day); %^a^ – percentage of adolescents: who are overweight/obese (LR^1^), who reach the MVPA (LR^2^) recommendations, who meet the ST recommendations (LR^3^ or LR^4^); LR^1–4^ – the reference group is the cohort from 2002; °gender comparison of prevalence of overweight/obesity, MVPA, and ST recommendations between groups of children with low and high FA (chi-square); ^#/^*p < 0.05, ^##/^**p < 0.01, ^###/^***p < 0.001

Regarding 60 or more minutes of MVPA daily, similar gender-related trend patterns were found between 2002 and 2014. However, different trend-related patterns of MVPA were detected in different FA category of adolescents. Except for adolescents from the high-FA category, for all the other boys and girls, a significant decrease in the achievement of ≥60 min of daily MVPA was revealed. However, in the percentage of achievement of MVPA recommendations in the last cycle of data collection (2013/2014), we discovered similar differences between adolescents from the low- and high-FA categories.

In 2014, the adolescents in the high-FA category show a significantly higher proportion in both MVPA than the adolescents in the low-FA category (>6 p.p) (Table 2). While FA-related differences are apparent in MVPA trend patterns between 2002 and 2014, a significant (*p* < 0.001) decrease in non-excessive ST (>10%) is obvious in the case of ST between 2002 and 2014, irrespective of gender, FA category, and the type of day of the week (Table 2). The most noticeable decline in non-excessive ST (≈20 p.p.) was found in low- and high-FA girls at weekends. However, when differences in non-excessive ST among the adolescents from the low- and high-FA categories were compared, gender-related differences were found between 2002 and 2014. While in the boys, the difference in non-excessive ST was gradually mitigating on weekdays (low FA-high FA: 8.8 p.p. 2002, 6.5 p.p. 2006, 3.7 p.p. 2010 and 3.0 p.p. 2014), the difference in non-excessive ST at weekends gradually increased in the girls (low FA-high FA: 2.3 p.p. 2002, 2.4 p.p. 2006, 3.2 p.p. 2010 and 4.5 p.p. 2014) (Table 2). In the last year of data collection, in 2014, more than 90% of the boys and more than 80% of the girls spent more than two hours per weekend day on ST, compared to 2002, when less than 80% of the boys and less than 68% of the girls exceeded the threshold of excessive ST. In addition, in 2014 the relative rates of boys reporting non-excessive ST in all the FA categories were barely half compared with the girls’ rates in, both on weekdays and at weekends (Table 2).

### Correlates of overweight/obesity in relation to family affluence

We found that girls and older adolescents had lower odds of overweight/obesity than boys and 11-year-old schoolchildren, respectively, in all the FA categories. However, the MVPA and ST correlates of adolescents’ overweight/obesity varied according to different FA categories. In the high-FA category of adolescents, achieving 60 min of MVPA daily or the absence of excessive ST on weekdays significantly reduced their odds of overweight/obesity. In contrast, only non-excessive ST at weekends was significantly associated with lower odds of overweight/obesity in the low-FA category of adolescents (Table 3). The differences in the odds of the prevalence of overweight/obesity in adolescents from distinct FA categories are obvious with regard to the year of data collection too. There were no significant differences in the odds of overweight/obesity in 2006 and 2010 in the low-FA category of adolescents, compared with 2002. However, a significant increase in the odds of their overweight/obesity was reported in 2014. In adolescents from the medium- and high-FA categories, there were significantly higher odds of overweight/obesity in all the data collection years (2006, 2010, and 2014) compared to the reference year 2002, but the odds of overweight/obesity in adolescents from the medium- and high-FA categories were somewhat lower in the last year of data collection (2014) than in 2006 and 2010 (Table 3).

**Table 3:** Correlates of overweight/obesity in randomized sample of Czech adolescents in relation to FA: HBSC survey 2002–2014

				Overweight/Obesity					
	Low FA			Medium FA			High FA		
		95%	CI		95%	CI		95%	CI
	OR	Lower	Upper	OR	Lower	Upper	OR	Lower	Upper
Year of data collection									
2002	Ref.			Ref.			Ref.		
2006	1.20	0.98	1.47	1.84***	1.57	2.16	1.76**	1.20	2.58
2010	1.18	0.91	1.53	1.71***	1.45	2.02	1.98***	1.38	2.84
2014	1.41***	1.17	1.70	1.63***	1.38	1.93	1.50*	1.03	2.19
Gender									
Boys	Ref.			Ref.			Ref.		
Girls	0.52***	0.45	0.61	0.44***	0.39	0.49	0.41***	0.34	0.50
Categorical age									
11 years	Ref.			Ref.			Ref.		
13 years	0.70***	0.59	0.84	0.70***	0.61	0.80	0.78*	0.62	0.98
15 years	0.52***	0.43	0.63	0.58***	0.51	0.67	0.63***	0.50	0.81
MVPA recommendation									
< 60 min of MVPA per day	Ref.			Ref.			Ref.		
≥ 60 min of MVPA per day	0.83	0.69	1.00	0.83**	0.73	0.95	0.69**	0.56	0.86
ST recommendations weekdays									
> 2 h per weekday	Ref.			Ref.			Ref.		
≤ 2 h per weekday	0.86	0.70	1.06	0.92	0.79	1.08	0.61**	0.44	0.84
ST recommendations weekend									
> 2 h per weekend day	Ref.			Ref.			Ref.		
≤ 2 h per weekend day	0.77*	0.61	0.96	0.94	0.79	1.11	1.04	0.74	1.45

*FA* family affluence, *MVPA* moderate-to-vigorous physical activity, *ST* screen time, *OR* odds ratio (logistic regression Enter method – three analyses separately for each category of FA); CI – 95% confidence interval; *p < 0.05, **p < 0.01, ***p < 0.001

## Discussion

Using nationally representative data collected over the 12-year study period, we assessed the trends in overweight/obesity and the PA and ST of 11–15-year-old adolescents in relation to the material affluence of their families. The results presented here indicate complex behavioural patterns that vary across FA categories. Obviously, there are increasing differences in the prevalence of overweight/obesity when comparing low-FA and high-FA boys between 2002 and 2014. Furthermore, a more pronounced decrease in achieving 60 min of MVPA daily is seen in adolescents from the low-FA category than adolescents from the high-FA category between 2002 and 2014.

### Overweight/obesity

In contrast with the economically developed countries of Western Europe and North America [32, 44], we observed an increase in the prevalence of overweight/obesity in the Czech adolescents across the FA categories between 2002 and 2014. Furthermore, it is disturbing that our findings reveal higher odds of overweight/obesity among 11-year-old adolescents than in their older peers, regardless of FA categories. In the economically developed countries of Western Europe and North America the opposite age-related pattern in terms of the prevalence of overweight/obesity generally prevails [32, 44]. The highest prevalence of overweight/obesity in low-FA girls and the highest increase in the trend-related incidence of overweight/obesity in low-FA boys might be related to unhealthy eating habits [14] and non-uniform socioeconomic development across all FA categories in low- and middle-income European countries [59, 60]. In addition, other lifestyle risk factors (smoking, alcohol consumption, cannabis use, and poor diet, especially sugar-sweetened beverages) seem to play their roles in the development of obesity in school-age children and adolescents [10, 59]. In the light of this information, it should also be noted that the Czech Republic, as the most affluent country of all of the Visegrad countries, also has the highest rate of adolescent risk behaviours (smoking, alcohol consumption, and cannabis use) [59].

### Moderate-to-vigorous physical activity

Different trend-related patterns in MVPA among 11-to-15-year-old adolescents in relation to FA were found between 2002 and 2014. While in the MVPA trends gender-related differences are not apparent, MVPA trends differ according to the FA categories. Similarly to most HBSC countries [44], a decrease in daily MVPA (represented ≥60 min per day) with increasing age was also found in adolescent Czech boys and girls in 2014. In 2014, a significantly higher proportion of Czech adolescents from the highest FA category reached the MVPA recommendation and showed a lower prevalence of overweight/obesity compared with the lowest FA category. Unlike a previous study [27], we found differences in MVPA between adolescents from the low- and high-FA categories. The countries without noticeable FA-related differences in MVPA include Austria, Belgium (French), Greenland, Ireland, Malta, Portugal, the Republic of Moldova, and Romania [44].

### Screen time

In line with previous studies [33, 35, 61], we found higher ST in adolescents of both genders at weekends than on weekdays, and overall higher ST in the boys than in the girls on both weekdays and weekend days. Similarly to adolescents from Canada, Iceland, Scotland, Finland, Germany, Italy, Slovenia, and Malta, girls of all ages reported less excessive ST than boys in 2014 [16, 44]. This finding probably reflects the natural higher need for face-to-face communication and social interaction in girls and the higher preference for PC gaming in boys [62]. However, unlike other studies [35, 63], the Czech adolescents from the low-FA category reported a slightly lower incidence of excessive ST (>2 h a day) than adolescents from the high-FA category both on weekdays and at weekends in all the data collection years between 2002 and 2014. This may partly be attributed to differences in rates of PC ownership between low-income households and high-income households [64]. However, further research on the topic is warranted because such a finding is rather unprecedented.

### Correlates of overweight/obesity in relation to family affluence

A previous trend-related study [16] revealed that the daily achievement of 60 min of MVPA and non-excessive ST on school days reduces the likelihood of the occurrence of overweight/obesity in Czech adolescents overall. However, the study did not analyse whether or how overweight/obesity correlates fluctuate among adolescent groups according to FA. In line with other studies [11, 20], different correlates of overweight/obesity have been revealed in different FA categories of adolescents. However, the above-mentioned comprehensive studies did not analyse the potential PA and ST correlates of overweight/obesity. Because child obesity is the result of a long-term adverse impact on the energy balance [65], it sounds logical that high-risk TV behaviours (excessive daily TV viewing, having a television in children’s bedrooms, and TV viewing during meals – [66, 67]) and low PA [15, 19] are directly associated with childhood overweight/obesity. However, in our study both correlates (non-excessive ST and sufficient PA) together significantly reduce the odds of overweight/obesity only in adolescents from the high-FA category. Sufficient PA reduces the risk of overweight/obesity in adolescents from the medium category of FA regardless of excessive ST but not in adolescents from the low-FA category. A higher overall volume of PA seems to be a stronger correlate of normal body weight than sedentary behaviour, because a higher level of MVPA measured with an accelerometer (high tertile groups of minutes of MVPA) among children and adolescents was associated with better cardiometabolic risk factors (waist circumference, fasting triglycerides, high-density lipoprotein cholesterol), regardless of their amount of sedentary time [68, 69]. Other relevant studies using accelerometers for PA measurement also find that higher MVPA (top versus the bottom quintiles of minutes of MVPA [70]) are strongly inversely correlated with obesity. In addition, higher levels of PA (the top versus the bottom quartiles of accelerometer counts per day) are prospectively associated with lower levels of obesity in adolescent white girls [71]. Although there are locally successful school-based PA interventions in the Czech Republic aimed at reducing overweight/obesity [72, 73], efforts to reduce the excessive body weight of schoolchildren continue to fail at the national level.

### Strengths and limitations

The findings from the present trend analysis need to be considered in the light of the methods used. The same standardized sampling procedure and data collection and management in all four survey cycles are essential prerequisites for conducting comparable trend studies across families with different levels of affluence. Although the level of BMI is generally considered as applicable for the estimation of the prevalence of overweight/obesity [74], it has been documented that overweight/obese adolescents tend to perceive themselves as being of about normal body weight [75, 76]. Social desirability can potentially affect self-reported data on body weight and height, as well as data on PA and ST; however, the magnitude, direction and trend-related variations of these potential changes remain unknown. In the HBSC study adolescents are assured of confidentiality and anonymity, which could have helped minimize the effect of social desirability in the participants’ responses. The FA scale was shown to be a valid tool for classifying the families of adolescents according to their material affluence [53, 55–58], but the FA scale does not capture whether the property inquired about is family-owned or subject to a mortgage. Although the SES of families may be classified as high by the FA scale, family finances spent on a mortgage can limit the expenses associated with physically active pastimes of adolescents or the daily consumption of fruit and vegetables.

### Future studies

A PA-friendly environment in the place of residence (available walking destinations and services, access to open space, e.g., parks, trails, green spaces) and school-related activity (school sports, physical education, and supervised PA) are positive correlates of PA in overweight and non-overweight children and adolescents [77–79], and a potential factor contributing to the lower incidence of overweight/obesity [72, 73]. Therefore, future trend studies should clarify the role of the school and residential environment in the changes over time in the prevalence of overweight/obesity and achieving the recommendations for PA and non-excessive ST.

## Conclusions

Overall, FA is a strong factor determining trend-related changes in the prevalence of overweight/obesity and PA patterns among Czech adolescents between 2002 and 2014. From the public health perspective, it is alarming that an increase of more than 10 p.p. in daily excessive screen time among adolescents in the low-FA category between 2002 and 2014 is also accompanied by a decrease of more than 5 p.p. in the achievement of 60 min of MVPA daily. The highest prevalence of overweight/obesity in schoolchildren from families with low levels of affluence makes it essential to develop more balanced intervention programmes for effective mitigation of childhood overweight and obesity.

## Abbreviations

BMI: Body Mass Index
CI: Confidence interval
FA: Family affluence
HBSC: Health Behaviour in School-aged Children
ICC: Intra class correlation
LR: Logistic regression
MVPA: Moderate-to-vigorous physical activity
OR: Odds ratio
PA: Physical activity
PC: Computer use
Ref.: Reference group
SES: Socioeconomic status
SPSS: Statistical Package for the Social Sciences
ST: Screen time
TV: Television viewing
WHO: World Health Organization
